# An automated tuberculosis screening strategy combining X-ray-based computer-aided detection and clinical information

**DOI:** 10.1038/srep25265

**Published:** 2016-04-29

**Authors:** Jaime Melendez, Clara I. Sánchez, Rick H. H. M. Philipsen, Pragnya Maduskar, Rodney Dawson, Grant Theron, Keertan Dheda, Bram van Ginneken

**Affiliations:** 1 Department of Radiology and Nuclear Medicine, Radboud university medical center, Nijmegen, Gelderland, the Netherlands; 2 Lung Infection and Immunity Unit, Division of Pulmonology and UCT Lung Institute, University of Cape Town, Cape Town, Western Cape, South Africa; 3 DST/NRF of Excellence for Biomedical Tuberculosis Research, and MRC Centre for Molecular and Cellular Biology, Division of Molecular Biology and Human Genetics, Faculty of Medicine and Health Sciences, Stellenbosch University, Tygerberg, South Africa

## Abstract

Lack of human resources and radiological interpretation expertise impair tuberculosis (TB) screening programmes in TB-endemic countries. Computer-aided detection (CAD) constitutes a viable alternative for chest radiograph (CXR) reading. However, no automated techniques that exploit the additional clinical information typically available during screening exist. To address this issue and optimally exploit this information, a machine learning-based combination framework is introduced. We have evaluated this framework on a database containing 392 patient records from suspected TB subjects prospectively recruited in Cape Town, South Africa. Each record comprised a CAD score, automatically computed from a CXR, and 12 clinical features. Comparisons with strategies relying on either CAD scores or clinical information alone were performed. Our results indicate that the combination framework outperforms the individual strategies in terms of the area under the receiving operating characteristic curve (0.84 versus 0.78 and 0.72), specificity at 95% sensitivity (49% versus 24% and 31%) and negative predictive value (98% versus 95% and 96%). Thus, it is believed that combining CAD and clinical information to estimate the risk of active disease is a promising tool for TB screening.

Tuberculosis (TB) is a leading cause of mortality and a major health problem worldwide. It is estimated that 9 million new TB cases and 1.5 million deaths due to TB occurred in 2013^1^. To slow down TB’s dissemination and reduce the risk and consequences of delayed treatment, the World Health Organization (WHO) recommends systematic screening of several risk groups, including household contacts and people living with HIV^2^. In this context, screening is defined as the “systematic identification, in a predetermined target group, of people with suspected active TB, by the application of tests, examinations, or other procedures which can be applied rapidly”^2^. To maximize the outcome of screening according to a specific goal, appropriate tests and algorithms must be carefully selected^3^,^4^.

A widely utilized test in TB screening is chest radiography. For this test, a posterior anterior chest radiograph (CXR) is obtained and examined either for abnormalities suggestive of TB or for any kind of abnormality. Chest radiography has been reported to yield high sensitivity and moderate to high specificity^3^. However, to achieve this performance level, expert readers are required. This is a major limitation, as in many high-TB-burden countries those experts are not sufficiently available^5^. Fortunately, with the introduction of digital CXR, computer-aided detection (CAD) has become an alternative to human reading. As shown in recent studies^6^,^7^, current CAD systems for CXR-based TB detection can perform at a comparable or superior level to clinical officers, which are the kind of personnel typically deployed under screening settings. Moreover, CAD may alleviate other issues related to CXR interpretation, such as high intra- and inter-observer variability^3^,^8^.

Symptom information is also used in TB screening algorithms. Symptoms suggestive of pulmonary TB include persistent cough, haemoptysis, weight loss, night sweats, etc. In general, symptom screening is considered less accurate and more variable than CXR screening^3^; therefore, symptom information may be more useful if paired with CXR^2^,^3^. Two basic approaches are possible: symptom information could be applied sequentially, i.e., as a prescreening test before CXR, or in parallel, i.e., both CXR and symptom assessment are used to decide if additional examination is needed. While the former approach is part of the WHO’s screening recommendations^2^, the latter is the preferred option in prevalence surveys, as it maximizes sensitivity^9^,^10^. As with the previous test, expertise is required to appropriately combine the cues provided by symptoms with the ones provided by CXR, and so a similar limitation is experienced. However, contrary to CXR testing, where CAD constitutes a potential solution, no automated approach has been devised to tackle the CXR/symptom fusion problem.

This paper proposes a novel strategy to combine automatic CXR scoring by CAD with clinical information, including symptoms and HIV status. We follow a similar approach to the parallel combination, but utilize machine-learning techniques to select the most relevant among the available inputs and fuse the selected inputs in an optimal way according to the specific characteristics of the data. Given that CXR screening is more accurate than symptom screening, our strategy starts with CXR scoring by CAD as its basis and attempts to improve its performance by means of clinical information. To assess the advantages of this approach, we compare its performance with that of alternative strategies relying either on CAD scores or clinical information only, where the latter also applies the proposed fusion by machine-learning techniques but excludes the CXR CAD score.

## Methods

### Data

The experimentation carried out in this study considers data used by Theron *et al.*^11^, who conducted a multicenter randomized controlled trial to assess Xpert MTB/RIF testing at primary care health centres in Southern Africa. The trial prospectively and consecutively enrolled subjects aged 18 years or above who presented to selected peri-urban clinics with one or more symptoms of TB according to predefined WHO criteria^12^, were able to spontaneously expectorate two sputum specimens, and had not received anti-tuberculosis treatment within the previous 60 days. Subjects who did not meet these criteria were excluded. Recruiting took place between 12 April 2011 and 30 March 2012. The study was carried out according to the protocol approved by the University of Cape Town Faculty of Health Sciences Research Ethics Committee (#404/2010). All the enrolled subjects provided written informed consent. Further details can be found in the parent study^11^.

In total, 392 patient records were available in our database. All these data were collected at Gugulethu TB Clinic (Cape Town, South Africa). Of the 392 patients, 73 were diagnosed with TB. The reference standard was culture positivity for Mycobacterium tuberculosis complex. Liquid culture (Mycobacteria Growth Indicator Tube, BD Microbiology Systems, Cockeysville, MD, USA) was performed on decontaminated sputum samples. A digital CXR was obtained from each patient with an Odelca DR unit (Delft Imaging Systems, Veenendaal, the Netherlands). All CXRs were processed by CAD4TB version 3.07^7^,^13^. Each of the patient records consisted of a CAD score in the range 0*–*100 and 12 clinical features. The clinical features were: body mass index (BMI), axillary temperature, heart rate, mid-upper arm circumference (MUAC), HIV status, anaemic conjunctivae, lung auscultation findings (any one of crepitation, rhonchi, or subdued or completely absent respiratory sounds), cough, haemoptysis, night sweats, dyspnoea and chest pain. They mostly corresponded to the variables used to compute the well-known TBscore^14^. The last five features (or symptoms) were self-reported. The remaining features (with the exception of HIV status) were assessed by an experienced research nurse under the supervision of a medical officer. The nurse received training in evaluating these specific features prior to the study start. For processing, the CAD scores and the first four features were represented using continuous values, whereas the remaining features were encoded as 1 (affirmative) or 0 (negative). The data are described in detail in Table 1.

### Combining CAD scores and clinical information for TB screening

**Figure 1:**
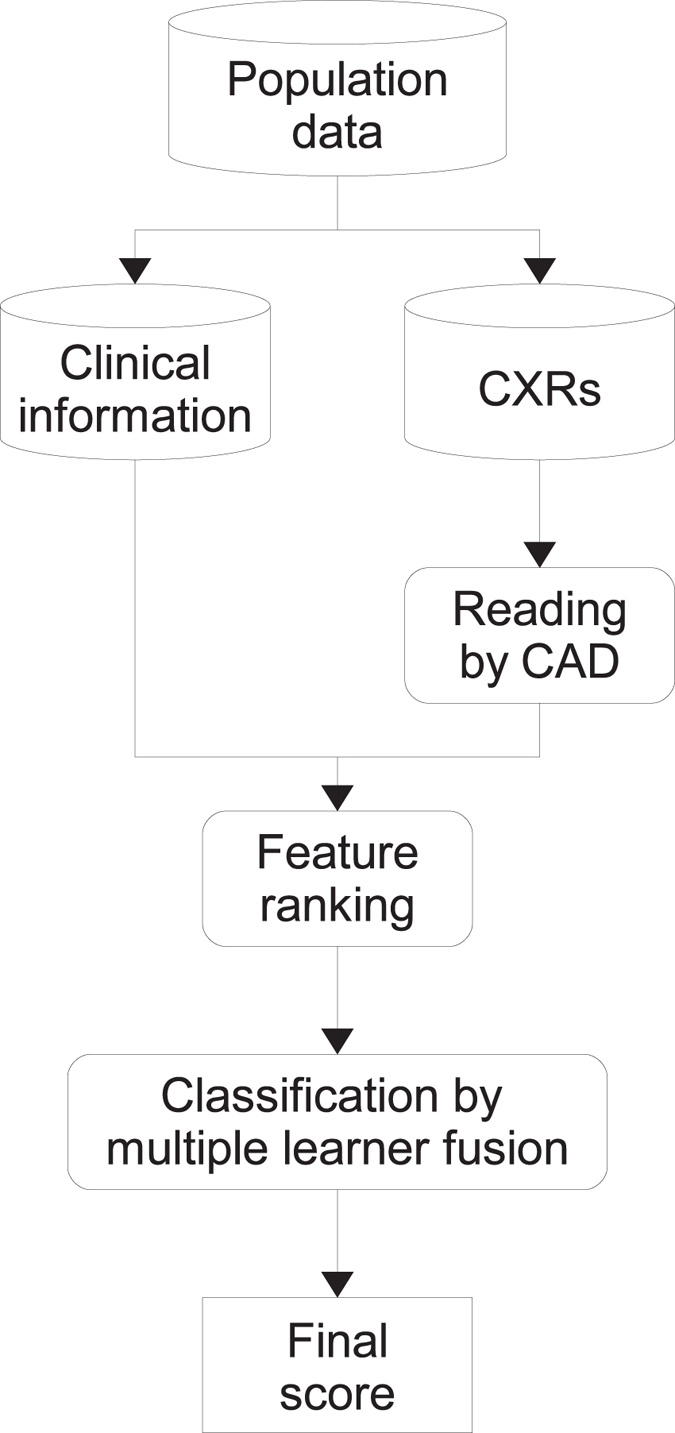
Framework for combining CAD scores and clinical information. CXR, chest radiograph; CAD, computer-aided detection.

The proposed framework for combining CAD scores and clinical information is shown in Fig. 1. It relies on two machine-learning procedures consisting in feature ranking and classification by multiple learner fusion. The interplay between these two components is the key to attain the sought improved performance considering the different sources of information. In the next sections, a detailed description of both components is provided.

### Feature ranking

Since not all the available features may be equally relevant for classification, and some of them may be even meaningless, determining which features can contribute the most is a crucial step in the proposed approach. To fulfil this task, we utilize the minimum redundancy maximum relevance (mRMR) algorithm^15^, which can assess the individual performance of a feature while taking into account its relation with the remaining ones. This is an important property, as it has been observed that combining the best individual features does not necessarily lead to the best performance^16^. In addition, being a “filter” method, mRMR ranks features independently of the classifier to be used, which is suitable for our combination framework, as it includes different classification techniques.

Given a supervised classification problem, mRMR attempts to identify those features that have the largest joint dependence on the target class. This is accomplished by optimizing the maximum relevance criterion defined by


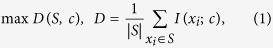


where *S* is the set of available features *x*_*i*_, *i* = 1, …, *M*; *c* is the target class and *I*(:) is the mutual information measure. Since it is likely that the top features ranked in this way will highly depend on each other, mRMR also adds the following minimum redundancy criterion to prioritize mutually exclusive features:


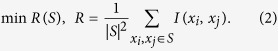


To combine the above criteria, an operator Φ(*D*, *R*) is defined and optimized according to


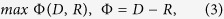


which leads to the feature ranking output by the method.

### Classification by multiple learner fusion

The rationale behind combining multiple learners is that different learning designs potentially provide complementary information that can improve classification accuracy^17^. Moreover, this mimics the approach followed by humans when critical decisions must be taken and thus a panel of experts, instead of a single specialist, is consulted. In medicine, for instance, combining the opinions of several specialists is common practice, and even combining the minimum amount of specialists (i.e., double reading) can lead to increased detection rates^18^,^19^. In consequence, the learner fusion strategy adopted in our framework is not only motivated by machine-learning theory but also by important findings in the field in which it would be applied.

In analogy to the double reading paradigm mentioned above, two state-of-the-art classification techniques are considered for fusion in our framework: random forests (RF)^20^ and extremely randomized trees (ERT)^21^. Although the foundation of RF and ERT is similar in the sense that both are ensembles of tree predictors and their learning algorithms heavily rely on randomization, the specific procedures that are subject to randomization is what sets the difference between these methods. While in the case of RF, randomization affects both the training set used to grow each tree predictor and the subset of features explored at each tree node to determine the best split, in the case of ERT, the node split itself (i.e., a feature index and a feature splitting value) is randomly selected. Therefore, the aforementioned procedures may be regarded as complementary ways of exploring the feature space.

To further take advantage of the utilized RF and ERT, feature selection based on the ranking provided by mRMR is carried out. In particular, we adopt a sequential forward selection approach, which aims at obtaining a near-optimal feature subset by sequentially adding each feature in the ranking to the ones already explored (those above in the ranking). To evaluate the goodness of the resulting feature subsets, each learner is applied independently. In the end, the selected feature subsets correspond to those that achieve the highest performance.

Besides the fused components, the fusion mechanism must be defined. Although this kind of fusion can be achieved by simple static rules, such as taking the sum, the product, the minimum or the maximum of the individual outputs^17^, we believe that a more sophisticated approach, such as a higher-level learner, may lead to a more refined set of rules and thus to a better performance. In consequence, we use a second ERT to carry out this task (we have observed in pilot experiments that ERT leads to better results than RF at this level).

### Learner Optimization

In order to obtain the best classification performance, we optimize two parameters shared by both RF and ERT. These parameters are the number and the maximum depth of the trees in the ensemble. Regarding the first parameter, it is well known that for this type of randomized approaches the prediction error is a monotonically decreasing function of the number of trees that stabilizes at a certain point^20^,^21^. Therefore, the choice of an appropriate number of trees essentially corresponds to determining the convergence point given the particularities of the classification problem. Regarding the second parameter, it is also well known that the deeper a tree, the more complex the data relations it can establish but, at the same time, the more prone to overfitting. Although it has been argued that trees in RF and ERT should be grown to their full extent (with no pruning)^20^,^21^, we have empirically observed that fine tuning this parameter results in improved performance.

To select appropriate values for the aforementioned parameters, we utilize a grid search strategy considering the training data. We explore values between 500 and 2000 trees, and 500 and 5000 trees for RF and ERT respectively. For the maximum tree depth, the points in the grid are heuristically set at 1, *M*/2, *M* and the full extent, with *M* being the number of available features. A slightly different setting is used for the ERT performing learner fusion. In this case, due to the reduced number of processed inputs (only two), trees with a maximum depth of 1, *M*, 2*M* and the full extent are explored.

### Evaluation

The performance of the compared strategies was assessed through 10-fold cross-validation. Under this setting, one tenth of the data was reserved for testing, and the remaining nine tenths were used for training, which included the feature ranking and learning procedures described in the preceding sections. Training and classification were carried out 10 times, each time with different folds, and once the 10 folds were processed, the classification scores assigned to the 10 test sets were pooled, and case-based receiver operating characteristic (ROC) analysis was conducted. The area under the raw ROC curve (AUC) was used as the main performance measure both during training and testing. Additional measures corresponding to the specificity at 95% sensitivity and the negative predictive value (NPV) were also computed, although for testing purposes only.

Statistical significance of the performance difference between pairs of compared approaches, as well as 95% confidence intervals (CIs), were determined by means of bootstrapping^22^,^23^. Cases were resampled with replacement in such a way that every bootstrap sample had the same number of cases as the original data set. For each new sample, two ROC curves corresponding to the two methods being compared were constructed, and the three performance measures mentioned above were computed. The p-values were defined as the fraction of performance differences that were negative or zero. The significance level was 0.05. The 95% CI was defined by the values bounding the 2.5% and 97.5% of the ordered data. The number of bootstrap samples was 5000.

## Results

The AUC achieved by the strategy combining CAD scores and clinical information was 0.84, whereas the AUCs for the alternative approaches using either type of information alone were 0.78 and 0.72 respectively. The difference between the combined strategy and the alternative approaches was significant (p = 0.0098 and p < 0.0002 respectively). At 95% sensitivity, combining the available cues resulted in 49% specificity and 98% NPV, whereas using CAD scores and clinical information on their own resulted in lower specificities and NPVs of 24% and 95%, and 31% and 96% for each approach. While the differences in specificity and NPV with respect to using CAD scores alone were significant (p = 0.0054 and p = 0.0154 respectively), only the difference in specificity was significant when comparing with the clinical information-based strategy (p = 0.0358; p = 0.1136 for NPV). There were no significant performance differences between the approaches using a single type of information. Results of all comparisons are listed in Table 2.

**Figure 2:**
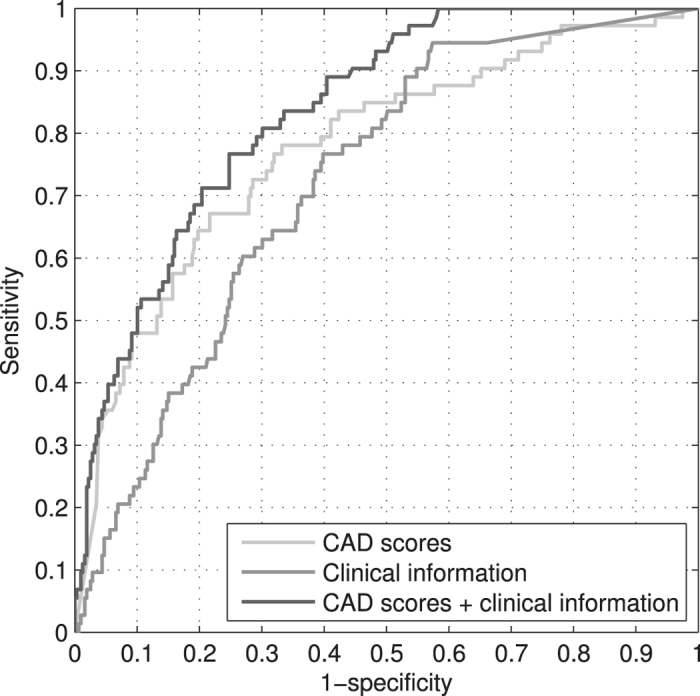
ROC curves yielded by the approaches evaluated in this study. The AUC for the proposed combined strategy is 0.84, whereas the AUCs for the strategies based on either CAD scores or clinical information are 0.78 and 0.72 respectively. ROC, receiving operating characteristic; AUC, area under the ROC curve; CAD, computer-aided detection.

The ROC curves associated with the above assessment are shown in Fig. 2. As can be seen, there is an increasing gap between the curves yielded by the combined strategy and CAD, which becomes more evident as specificity decreases to its minimum. When comparing the combined strategy with the one using clinical information only, the difference is larger for most of the curve, including zones of high specificity. Moreover, while the combined strategy reaches perfect sensitivity at more than 40% specificity, the other two strategies attain this level of sensitivity only at near-zero specificity.

Besides the final output of our framework, the data produced by its intermediate steps also provide valuable insights into our research topic. For example, a key issue is identifying which features are the most relevant given our setting. Rankings indicating the relevance of the available features are shown in Supplementary Tables S1 and S2. The highest AUC, which corresponds to the optimal set of features, is shown in bold. In terms of feature ranking, there is a marked difference between the combined and clinical information-based strategies, with CAD score being the most relevant feature for the former and heart rate being the most relevant feature for the latter. In terms of feature selection, the difference resides in the number of selected features. In this regard, the learners of the combined strategy require less information (five and four features) than the learners of the clinical information-based strategy (eight and six features) to reach their optimal performance.

## Discussion

The main findings of our study are summarized below and discussed in the subsequent paragraphs.

1. The combination of CAD and clinical information offers improved accuracy and increased specificity compared with using either type of information on its own.
2. When applied as a hypothetical rule-out test for TB with a sensitivity of 95%, the specificity and NPV of the combined approach are 49% and 98% respectively.
3. It is easier to reach optimal performance in the presence of CAD scores. In this context, and given our data, the most valuable clinical features are HIV status, axillary temperature and lung auscultation findings.

As indicated above, the automated strategy combining CAD scores and clinical information leads to a substantial improvement over the alternative approaches that use each type of information separately. When comparing with the CAD scores-based strategy, we observe from the ROC curves that the additional information is mostly beneficial at medium to low specificity, which is to be expected, as the cases associated with these zones of the curve are those with increasing difficulty in terms of radiological assessment. In this situation, the additional cues provided by the clinical information compensate for the absence of a correct outcome from CAD, which is generally due to subtle, small or unusual TB manifestations, or a completely normal CXR appearance. Supplementary Fig. S1a–d illustrate these cases, where an improvement in abnormality ranks (which are derived from the scores of each approach sorted in decreasing order to enable per-case comparisons) can be noticed. In the same line, a substantial gap between the ROCs associated with the combined approach and the strategy based on clinical information is observed. This time, however, the difference is evident even at high specificity, which is probably due to the discriminative power of CXR. Related examples are shown in Supplementary Fig. S1e,f.

Besides the aggregate analysis of the curves, evaluation at 95% sensitivity shows that our combined strategy attains a specificity of 49%, whereas the CAD-based and the clinical information-based strategies reach specificities of 24% and 31% respectively (details on the number of cases wrongly classified by the individual strategies but correctly classified by the combined strategy and vice versa are given in Supplementary Table S3). This noticeable difference corresponds to a reduction of 33% and 26% in the number of false-positive detections compared with either strategy, suggesting that the combined method would be a better option for triage, which is the most likely intended application. In that case, subjects with a positive result would undergo further testing (e.g., culture or Xpert), while subjects with a negative result would require no further action. Although the specificity at 95% sensitivity is the suggested evaluation criterion for TB triage tests^24^, different operating conditions may exist, and thus a different cut-off point may be selected. According to our current results, the higher the required sensitivity, the higher the advantage of the combined strategy over the individual ones (see Fig. 2). On the contrary, the lower the sensitivity, the lesser the advantage, especially with respect to CAD, but as discussed before, that is a foreseen outcome considering the radiological evidence.

Examples of normal cases correctly labelled by our combined method but identified as TB suspects by the other strategies are shown in Supplementary Fig. S1g,h. In the first example, there is a suspicious pattern in the top part of the right lung that is strongly detected by CAD; however, only a few symptoms: cough, night sweats and chest pain are reported, which leads the combined strategy to assign a low abnormality score. In the second case, although more clinical signs, including an HIV-positive status, are present, other relevant features, such as axillary temperature and lung auscultation findings, have normal values, and the CXR score is low. As a result, the final abnormality score is also low. Unfortunately, trade-offs are inevitable when following a combined data-driven approach, and while most of the cases improve their ranks with respect to the ones assigned by the individual strategies, some instances, on the contrary, experience degradation. An example exhibiting this kind of degradation with respect to CAD is this same non-TB case shown in Supplementary Fig. S1h. Apparently, the feature driving the relatively high rank is HIV status. Since there is a high HIV prevalence among the TB cases in our data set (53.4%), the presence of this feature in this particular instance may have influenced our machine learners toward a not-too-accurate prediction. Examples of cases with degraded ranks with respect to the clinical information-based strategy can be seen in Supplementary Fig. S1a,b,d. In those cases, it seems that the radiological evidence needed to trigger a high abnormality rank outweighs the contribution of the clinical features according to the automatically inferred classification rules. Thus, despite the presence of HIV as emphasized above, the clinical information-based rank cannot be matched.

For the feature ranking and selection procedures, it is not surprising that CAD score is the most relevant feature for the combined strategy, as it is a complete screening strategy in its own right and leads to a much higher overall performance than the combination of the remaining features (which constitute the second alternative strategy). In addition, the lower number of features required by the learners of the combined strategy to reach their optimal performance and their substantially higher AUC values compared with the learners of the clinical information-based strategy indicate that it is easier to infer appropriate classification rules in the presence of CAD scores. In this sense, our approach is not only effective but also logical, as it takes the CAD score as its basis and then attempts to refine its discrimination power by adding complementary information. Furthermore, the low number of features added to this basis suggests that only a few clinical signs are needed to improve CAD/CXR performance, which has important practical benefits, such as facilitating the process of data collection itself, as not every possible test or examination would need to be performed. This, in turn, may lead to more agile logistics, as well as reduced costs.

Another notable result is that features such as HIV status, axillary temperature and lung auscultation findings were always included, which indicates that these features always provided additional cues to improve performance. On the other hand, cough, which is a symptom recommended for screening^2^,^25^, was ranked with medium to low importance only and was even disregarded for the combined strategy. This can be explained by the setting of our study: in passive case finding the vast majority of subjects cough (98.5% in our database), and therefore, the discriminative power of this feature is low. A final interesting point is that the selected features (excluding CAD score) mostly corresponded to outcomes of examination or testing instead of self-reported symptoms. This could be due to the more subjective nature of self-reported symptoms. Symptom self-reporting may also be affected by several other factors, such as linguistics and ethnic and cultural beliefs^26^. The feature selection mechanism included in our framework could help to minimize the effects of those factors by giving more relevance to the truly useful information.

One limitation of our study is the use of data from a single site. A second limitation is that only a passive case finding scenario was considered (all the enrolled subjects were TB suspects presenting to a clinic) and so the prevalence of TB was high. In this respect, further studies are needed to determine if a combined approach that fuses computerized CXR analysis with clinical information also improves performance in other populations and settings, such as active case finding and prevalence surveys.

In conclusion, machine-learning techniques can be used to optimally fuse the most relevant factors among the available information according to the characteristics of the given data. Our experiments have shown that the proposed combination framework improved the specificity of CAD and outperformed alternative approaches using CXR CAD scores or clinical information alone. We therefore believe that this new strategy is a promising tool for TB case finding in situations where automated screening is the most suitable or, perhaps, the only option.

## Additional Information

**How to cite this article**: Melendez, J. *et al.* An automated tuberculosis screening strategy combining X-ray-based computer-aided detection and clinical information. *Sci. Rep.* **6**, 25265; doi: 10.1038/srep25265 (2016).

## Supplementary Material

Supplementary Information

## Figures and Tables

**Table 1:** Demographics, symptoms and findings corresponding to the subjects included in the current study.

Characteristic	All subjects (n = 392)	TB cases (n = 73)	Non-TB cases (n = 319)	p-value*
	No. (%)	No. (%)	No. (%)	
Mean age (SD)	40 (11.9)	38 (11.5)	40 (12.0)	0.0849
Gender				0.6520
Female	152 (38.8)	30 (41.1)	122 (38.2)	
Male	240 (61.2)	43 (58.9)	197 (61.8)	
BMI < 18†				0.0047
Yes	36 (9.2)	13 (17.8)	23 (7.2)	
No	356 (90.8)	60 (82.2)	296 (92.8)	
Axillary temperature > 37 °C†				<0.0001
Yes	48 (12.2)	26 (35.6)	22 (6.9)	
No	344 (87.8)	47 (64.4)	297 (93.1)	
Heart rate > 90/min†				<0.0001
Yes	102 (26.0)	34 (46.6)	68 (21.3)	
No	290 (74.0)	39 (53.4)	251 (78.7)	
MUAC < 220 mm†				0.0010
Yes	36 (9.2)	14 (19.2)	22 (6.9)	
No	356 (90.8)	59 (80.8)	297 (93.1)	
Anaemic conjunctivae				0.7419
Yes	4 (1.0)	1 (1.4)	3 (0.9)	
No	388 (99.0)	72 (98.6)	316 (99.1)	
Lung auscultation findings				0.2310
Yes	217 (55.4)	45 (61.6)	172 (53.9)	
No	175 (44.6)	28 (38.4)	147 (46.1)	
Cough				0.9013
Yes	386 (98.5)	72 (98.6)	314 (98.4)	
No	6 (1.5)	1 (1.4)	5 (1.6)	
Haemoptysis				0.4620
Yes	49 (12.5)	11 (15.1)	38 (11.9)	
No	343 (87.5)	62 (84.1)	281 (88.1)	
Night sweats				0.9105
Yes	292 (74.5)	54 (74.0)	238 (74.6)	
No	100 (25.5)	19 (26.0)	81 (25.4)	
Dyspnoea				0.6857
Yes	137 (34.9)	27 (37.0)	110 (34.5)	
No	255 (65.1)	46 (63.0)	209 (65.5)	
Chest pain				0.8974
Yes	266 (67.9)	50 (68.5)	216 (67.7)	
No	126 (32.1)	23 (31.5)	103 (32.3)	
HIV status				<0.0001
Positive	130 (33.2)	39 (53.4)	91 (28.5)	
Negative	262 (66.8)	34 (46.6)	228 (71.5)	
CAD score > 60‡				<0.0001
Yes	240 (61.2)	63 (86.3)	177 (55.5)	
No	152 (38.8)	10 (13.7)	142 (44.5)	

^*^Significance testing was done using the chi-squared test, except for age, for which, due to its continuous nature, the t-test was utilized.

^†^Threshold set according to Wejse *et al.*^14^.

^‡^Threshold set according to Muyoyeta *et al.*^8^.

TB, tuberculosis; SD, standard deviation; BMI, body mass index; MUAC, mid-upper arm circumference; CAD, computer-aided detection.

**Table 2:** Performance of the evaluated screening strategies (AUC, specificity at 95% sensitivity and NPV) and the p-values obtained when comparing with the strategy using CAD scores only (vs. CAD scores) and the strategy using clinical information only (vs. clinical inform.).

Strategy	Performance								
	AUC	vs. CAD scores	vs. clinical inform.	Spec. at 95% sens.	vs. CAD scores	vs. clinical inform.	NPV	vs. CAD scores	vs. clinical inform.
CAD Scores	0.78 (0.71 to 0.84)	–	0.0978	24% (5% to 39%)	–	0.2906	95% (87% to 97%)	–	0.1792
Clinical information	0.72 (0.66 to 0.78)	0.0978	–	31% (15% to 49%)	0.2906	–	96% (93% to 98%)	0.1792	–
CAD scores + clinical information	0.84 (0.79 to 0.88)	0.0098	<0.0002	49% (40% to 60%)	0.0054	0.0358	98% (96% to 98%)	0.0154	0.1136

The 95% CIs are shown between parentheses.

Significant differences are shown in bold.

AUC, area under the ROC curve; ROC, receiving operating characteristic; CI, confidence interval; NPV, negative predictive value; CAD, computer-aided detection.
