# Supplementary material for: An automated tuberculosis screening strategy combining X-ray-based computer-aided detection and clinical information
Source: Sci Rep. 2016 Apr 29;6:25265. doi: 10.1038/srep25265 (PMC4850474; doi:10.1038/srep25265)
Supplement: Supplementary Information [file srep25265-s1.pdf]

Supplementary material

An automated tuberculosis screening strategy combining X-ray-based computer-aided detection and clinical information

Jaime Melendez, Clara I. Sánchez, Rick H. H. M. Philipsen, Pragnya Maduskar, Rodney Dawson, Grant Theron, Keertan Dheda, Bram van Ginneken

Supplementary Table S1 Feature ranking and average AUC (10-fold CV) after sequentially adding each feature considering the fused learners (RF and ERT) and combined data consisting of CAD scores and clinical information. The highest AUC, which corresponds to the optimal set of features for each learner, is shown in bold.

Rank	Feature	Average AUC RF	Average AUC ERT
1	CAD score	0.76	0.76
2	HIV status	0.81	0.78
3	Axillary temperature	0.82	0.80
4	Lung auscultation findings	0.82	**0.81**
5	Haemoptysis	**0.82**	0.80
6	Heart rate	0.81	0.81
7	MUAC	0.81	0.81
8	Night sweats	0.81	0.81
9	Anaemic conjunctivae	0.81	0.81
10	Cough	0.81	0.80
11	Chest pain	0.81	0.79
12	Dyspnoea	0.81	0.79
13	BMI	0.80	0.79

AUC, area under the ROC curve; ROC, receiving operating characteristic; CV, cross-validation; RF, random forest; ERT, extremely randomized trees; CAD, computer-aided detection; MUAC, mid-upper arm circumference; BMI, body mass index.

Supplementary Table S2 Feature ranking and average AUC (10-fold CV) after sequentially adding each feature considering the fused learners (RF and ERT) and clinical information. The highest AUC, which corresponds to the optimal set of features for each learner, is shown in bold.

Rank	Feature	Average AUC RF	Average AUC ERT
1	Heart rate	0.68	0.68
2	Anaemic conjunctivae	0.67	0.69
3	MUAC	0.70	0.73
4	HIV status	0.72	0.72
5	Lung auscultation findings	0.73	0.73
6	Axillary temperature	0.75	**0.74**
7	Haemoptysis	0.76	0.74
8	Cough	**0.76**	0.74
9	Night sweats	0.75	0.73
10	Chest pain	0.75	0.73
11	Dyspnoea	0.75	0.73
12	BMI	0.75	0.73

AUC, area under the ROC curve; ROC, receiving operating characteristic; CV, cross-validation; RF, random forest; ERT, extremely randomized trees; MUAC, mid-upper arm circumference; BMI, body mass index.

a

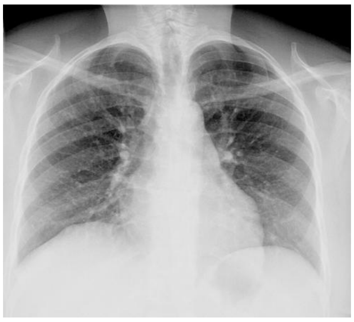

BMI:	35.0	Culture result:	positive
Axillary temperature:	36.5		
Heart rate:	116	Rank by CAD:	227/392
MUAC:	330		
Anaemic conjunctivae:	no	Rank by clinical	
Lung auscultation findings:	yes	information:	74/392
Cough:	yes		
Haemoptysis:	yes	Rank by CAD +	
Night sweats:	yes	clinical information:	134/392
Dyspnea:	no		
Chest pain:	yes		
HIV status:	positive		

b

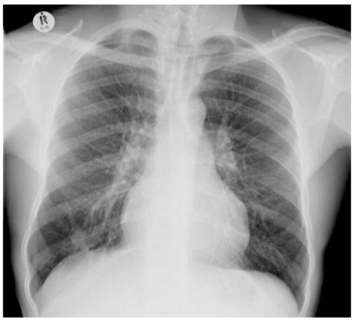

BMI:	21.9	Culture result:	positive
Axillary temperature:	39.4		
Heart rate:	124	Rank by CAD:	295/392
MUAC:	260		
Anaemic conjunctivae:	no	Rank by clinical	
Lung auscultation findings:	yes	information:	16/392
Cough:	yes		
Haemoptysis:	no	Rank by CAD +	
Night sweats:	yes	clinical information:	117/392
Dyspnea:	no		
Chest pain:	yes		
HIV status:	positive		

c

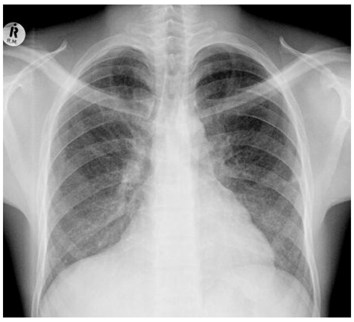

BMI:	19.4	Culture result:	positive
Axillary temperature:	38.2		
Heart rate:	110	Rank by CAD:	156/392
MUAC:	255		
Anaemic conjunctivae:	no	Rank by clinical	
Lung auscultation findings:	no	information:	27/392
Cough:	yes		
Haemoptysis:	no	Rank by CAD +	
Night sweats:	yes	clinical information:	27/392
Dyspnea:	no		
Chest pain:	yes		
HIV status:	positive		

d

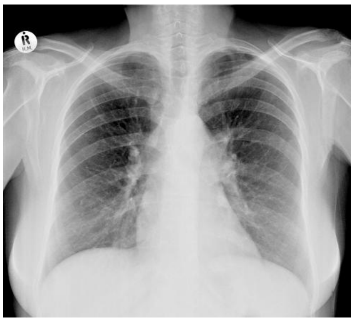

BMI:	23.9	Culture result:	positive
Axillary temperature:	36.8		
Heart rate:	110	Rank by CAD:	369/392
MUAC:	260		
Anaemic conjunctivae:	no	Rank by clinical	
Lung auscultation findings:	yes	information:	91/392
Cough:	yes		
Haemoptysis:	no	Rank by CAD +	
Night sweats:	yes	clinical information:	189/392
Dyspnea:	no		
Chest pain:	no		
HIV status:	positive		

Supplementary Figure S1 Examples of CXRs and their ranks as assigned by the evaluated screening strategies. (**a**) A TB case with a subtle fibrotic lesion (right lung, middle part). (**b**) A TB case with a conspicuous but small opacity (right lung, bottom part). (**c**) A TB case with rare manifestations (miliary pattern and lymphadenopathy). (**d**) A TB case with an apparently normal CXR. Note that the ranks assigned by the combined strategy are improved with respect to CAD (the higher the rank, the higher the likelihood of the CXR being abnormal), but in the first, second and fourth cases, degradation is experienced with respect to clinical information.
CXR, chest radiograph; CAD, computer-aided detection; BMI, body mass index; MUAC, mid-upper arm circumference; TB, tuberculosis.

e

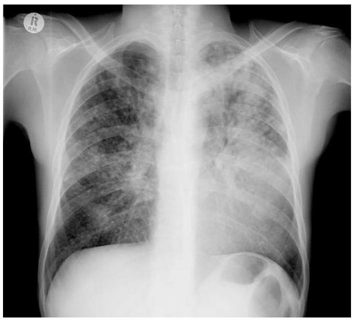

BMI:	14.1	Culture result:	positive
Axillary temperature:	35.6		
Heart rate:	99	Rank by CAD:	4/392
MUAC:	220		
Anaemic conjunctivae:	no	Rank by clinical information:	71/392
Lung auscultation findings:	no		
Cough:	yes		
Haemoptysis:	no	Rank by CAD + clinical information:	6/392
Night sweats:	no		
Dyspnea:	yes		
Chest pain:	no		
HIV status:	positive		

f

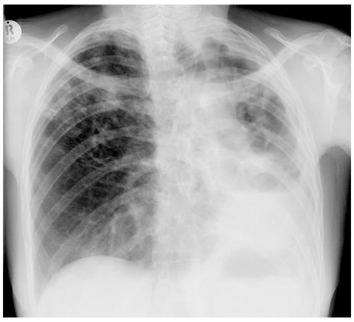

BMI:	20.0	Culture result:	positive
Axillary temperature:	35.9		
Heart rate:	89	Rank by CAD:	21/392
MUAC:	235		
Anaemic conjunctivae:	no	Rank by clinical information:	152/392
Lung auscultation findings:	no		
Cough:	yes		
Haemoptysis:	no	Rank by CAD + clinical information:	10/392
Night sweats:	yes		
Dyspnea:	yes		
Chest pain:	yes		
HIV status:	positive		

g

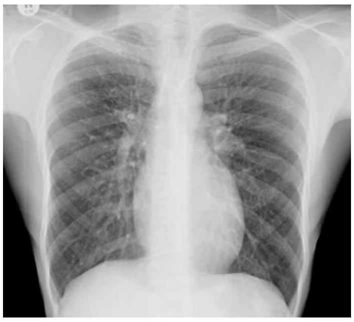

BMI:	18.5	Culture result:	negative
Axillary temperature:	36.4		
Heart rate:	61	Rank by CAD:	176/392
MUAC:	240		
Anaemic conjunctivae:	no	Rank by clinical information:	351/392
Lung auscultation findings:	no		
Cough:	yes		
Haemoptysis:	no	Rank by CAD + clinical information:	348/392
Night sweats:	yes		
Dyspnea:	no		
Chest pain:	yes		
HIV status:	negative		

h

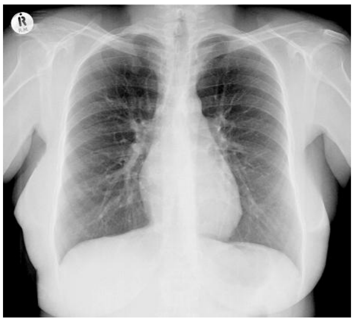

BMI:	24.7	Culture result:	negative
Axillary temperature:	35.3		
Heart rate:	86	Rank by CAD:	338/392
MUAC:	300		
Anaemic conjunctivae:	no	Rank by clinical information:	132/392
Lung auscultation findings:	no		
Cough:	yes		
Haemoptysis:	no	Rank by CAD + clinical information:	237/392
Night sweats:	yes		
Dyspnea:	yes		
Chest pain:	yes		
HIV status:	positive		

Supplementary Figure S1 (cont.) (**e**) A TB case with obvious lesions in both lungs ranked not particularly high by clinical information (the higher the rank the higher the likelihood of the CXR being abnormal). (**f**) A similar TB case with even a lower rank. (**g**) A non-TB case highly ranked by CAD due to a suspicious pattern in the top part of the right lung. (**h**) A non-TB case with a high rank due to clinical information. In the first two cases, the ranks assigned by both CAD and the combined strategy are considerably higher than the ranks assigned by means of clinical information. The last two cases correspond to false-positive detections by CAD and clinical information respectively, when using a rule-out threshold set at 95% sensitivity. The combined strategy, in contrast, correctly identifies these cases as negative and assigns lower ranks. Note, though, that rank degradation with respect to CAD is experienced in the last example. CXR, chest radiograph; CAD, computer-aided detection; BMI, body mass index; MUAC, mid-upper arm circumference; TB, tuberculosis.

Supplementary Table S3 Number of cases wrongly classified by the individual strategies but correctly classified by the combined strategy and vice versa. The cut-off point leading to 95% sensitivity was used to binarize the outputs.

	**No.**
FPs by CAD scores but TNs by the combined strategy	107
FNs by CAD scores but TPs by the combined strategy	4
FPs by the combined strategy but TNs by CAD scores	26
FNs by the combined strategy but TPs by CAD scores	4
FPs by clinical information but TNs by the combined strategy	78
FNs by clinical information but TPs by the combined strategy	2
FPs by the combined strategy but TNs by clinical information	20
FNs by the combined strategy but TPs by clinical information	2

FPs, false positives; TNs, true negatives; FNs, false negatives; TPs, true positives; CAD, computer-aided detection.
